# Minimally invasive approach in a rare emergency surgery, gallbladder perforation

**DOI:** 10.1186/s12893-024-02495-z

**Published:** 2024-07-10

**Authors:** Yunushan Furkan Aydoğdu, Emre Gülçek, Ahmet Can Koyuncuoğlu, Çağrı Büyükkasap, Kürşat Dikmen

**Affiliations:** 1 Department of General Surgery, Bandırma Training and Research Hospital, Balıkesir, Turkey; 2 Department of General Surgery, Polatlı Duatepe State Hospital, Ankara, Turkey; 3 Faculty of Medicine, Department of General Surgery, Gazi University, Ankara, Turkey

**Keywords:** Gallbladder perforation, Modified Niemeier type I, Minimally invasive surgery, Laparoscopy, Conversion to open cholecystectomy

## Abstract

**Background:**

Gallbladder perforations are challenging to manage for surgeons due to their high morbidity and mortality, rarity, and surgical approach. Laparoscopic cholecystectomy (LC) is now included with open cholecystectomy in surgical managing gallbladder perforations. This study aimed to evaluate the factors affecting conversion from laparoscopic to open cholecystectomy in cases of type I gallbladder perforation according to the Modified Niemeier classification.

**Methods:**

Patients who met the inclusion criteria were divided into two groups: LC and conversion to open cholecystectomy (COC). Demographic, clinical, radiologic, intraoperative, and postoperative factors were compared between groups.

**Results:**

This study included 42 patients who met the inclusion criteria, of which 28 were in the LC group and 14 were in the COC group. Their median age was 68 (55–85) years. Age did not differ significantly between groups (*p* = 0.218). However, the sex distribution did differ significantly between groups (*p* = 0.025). The location of the perforation differed significantly between groups (*p* < 0.001). In the LC group, 22 patients were perforated from the fundus, four from the trunk, and two from the neck. In the COC group, two patients were perforated from the fundus, four from the trunk, and eight from the neck. Surgical procedure times differed significantly between the LC (105.0 min [60–225]) and COC (125.0 min [110–180]) groups (*p* = 0.035). The age of the primary surgeons also differed significantly between the LC (42 years [34–63]) and COC (55 years [36–59]) groups (*p* = 0.001).

**Conclusions:**

LC can be safely performed for modified Niemeier type I gallbladder perforations. The proximity of the perforation site to Calot’s triangle, Charlson comorbidity index (CCI), and Tokyo classification are factors affecting conversion from laparoscopic to open surgery of gallbladder perforations.

## Introduction

Among surgical emergencies involving the gallbladder, perforation cases have higher morbidity and mortality rates, and preoperative evaluation is difficult for surgeons because of their rarity [1–3]. Niemeier published a classification system in his study on gallbladder perforations. According to the classification system, type I is chronic perforation with cholecystoenteric fistula, type II is subacute perforation with spreading abscess, and type III is acute perforation (into the free peritoneal cavity) [4]. Subsequently, Fletcher AG Jr et al. reclassified gallbladder perforations in their study. According to their classification, type I is acute, free perforation, type II subacute perforation with pericholecystic abscess, and type III chronic perforation with cholecystoenteric fistula [5]. Today, while surgical methods have changed, their classification system remains relevant. They grouped them into three types based on the components of the status of the septic picture and whether they are acute or chronic [6]. The type I group represents generalized biliary peritonitis, and cases almost always require emergency surgical intervention [6, 7]. Early surgery has a good prognosis. In type II perforation, cholecystostomy should be considered first and is effective in healing. Fletcher AG Jr et al. stated that cholecystectomy, fistula closure and, if necessary, choledochostomy should be considered in type III perforation [5]. However, when the current literature is examined, it is seen that there is no clear consensus on the treatment of cholecystoenteric fistula [8–10].

Laparoscopic cholecystectomy, a surgical procedure frequently performed by surgeons in routine care, is now included with open cholecystectomy in the surgery for gallbladder perforations [4, 11].

In addition to acute abdominal examination and pneumoperitoneum, which are the most important factors in the emergency surgery decision, abdominal computed tomography (CT) may guide surgeons when gallbladder perforation is suspected in patients with a positive Murphy’s sign. Abdominal CT is the examination of choice since it is specific and sensitive in showing gallbladder stones, air in its wall, and the pericholecystic fluid around it [12].

Clinical studies have identified surgical and clinical differences between modified Niemeier classification types [1, 6, 13]. To contribute to the literature, we aimed to compare the preoperative and postoperative clinical characteristics of patients with type I gallbladder perforations according to the modified Niemeier classification and requiring emergency surgery between those who underwent laparoscopic surgery and those who required conversion from laparoscopic to open surgery.

## Materials and methods

**Fig. 1:**
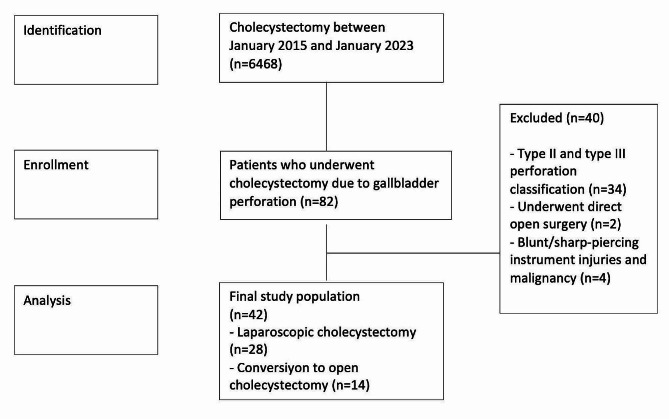
The sample collection scheme

This study initially available 6468 patients who underwent cholecystectomy in a tertiary health center in the last five years (between January 2018 and January 2023). Subsequently, 82 patients who were operated on due to gallbladder perforation were evaluated. 48 patients were classified as type I, 31 as type II, and 3 as type III. In the treatment of patients in the type II and type III groups, emergency surgery should not be applied in the foreground and elective surgery is required after follow-up. In this retrospective single-center experience study, retrospective data of patients with type II and type III perforation could not be objectively evaluated. Therefore, 36 patients who were not classified as modified Niemeier type I and underwent direct open surgery were excluded. Its inclusion criteria were diagnosis by radiologic imaging methods during emergency admission and/or laparoscopic surgery after confirmation of the gallbladder perforation diagnosis during surgery. Four patients with perforation due to blunt/sharp-piercing instrument injuries and malignancy were excluded. This study evaluated 42 patients who met the relevant criteria (Fig. 1). STROBE guidelines were followed while performing this study [14].

This study’s primary aim was to evaluate the effect of a minimally invasive approach on mortality and morbidity in patients with modified Niemeier type I gallbladder perforations requiring emergency surgery. Therefore, clinical outcomes were evaluated in detail and compared with preoperative comorbid conditions in the included patients.

We evaluated the radiological imaging methods used in the diagnosis, demographic characteristics, complaints at admission, ASA (American Society of Anesthesiologists) physical status classification system, Charlson comorbidity index [15], Tokyo Guidelines 2018 [16], Modified Nassar scales [17], preoperative white blood cell (WBC) and blood glucose values, preoperative vital signs, comorbid diseases, surgical approach, and surgery duration. We also evaluated postoperative complications, Clavien-Dindo classification [18], hospitalization duration, mortality, successful surgery completion, and intraoperative bile fluid cultures. Patients were followed up until postoperative day 30. We divided the included patients into two groups based on the surgical approach (laparoscopic and conversion from laparoscopic to open surgery) and compared the specified parameters between them.

### Surgical approach method

Laparoscopic cholecystectomy (standard 4 port): The operation was started under general anesthesia after appropriate field cleaning and sterile draping. After a mini transverse incision under the umbilicus, intraabdominal insufflation was achieved with a veress needle (the hasson technique was used in the presence of a previous operation and umbilical hernia). The abdomen was entered with an 11 mm trocar. Exploration was performed with a camera inserted through the trocar. It was seen that the biliary incision was perforated. Then 1 11 mm and 2 5 mm ports were inserted under direct vision and positioned. The gallbladder was suspended from the fundus with the help of a grasper and exposed. The sac was separated from the liver bed with blunt and sharp dissections and dissected up to Hartmann. Calot’s triangle was dissected and the cystic duct was reached. The cystic artery was found. Two clips were placed distal and one proximal to the duct and cut. The critical view of safety was obtained after dissection. One clip was placed on the cystic artery and it was cauterized distally and cut. The sac was separated from the liver bed with hook cautery and taken out of the abdomen. A free drain was placed in the lumen. Following bleeding control, the skin openings were closed one by one with 3/0 prolene (Doğsan™, Turkey), and the procedure was ended with dressing.

Conversion to open cholecystectomy: The abdomen was then entered through the right subcostal incision. The sac was separated from the liver bed with blunt and sharp dissections and dissected up to Hartmann. The cystic duct was reached by dissecting the Calot triangle. The cystic artery was found. The critical view of safety was obtained after dissection. Clips were placed and/or ligated distal and proximal to the duct, and then the cystic duct was cut. The cystic artery was clipped and/or ligated. It was cauterized and cut distally. The sac was separated from the liver bed with hook cautery and taken out of the abdomen. A free drain was placed in the lumen. Following bleeding control, the layers were closed one by one and the procedure was terminated with dressing.

Both surgical procedures were performed as described in the standard. In the conversion to open cholecystectomy group, intraoperative cholangiography was performed in 5 patients in whom the safety of the biliary tract was uncertain.

### Statistical analysis

Statistical analyses were performed using the Statistical Package for the Social Sciences software (version 26.0; SPSS Inc, Chicago, IL, USA). Descriptive statistics were utilized, presenting numerical values in the form of median (min-max), while categorical variables were expressed as frequency and percentage. Quantitative variables were compared between groups using Kruskal–Wallis or Mann–Whitney U tests. Qualitative variables were compared between groups using Pearson’s chi-square or Fisher’s exact tests. Fisher’s exact post-hoc test was used to assess the significance of differences in the anatomical location of the perforations. A multivariate regression model was performed to identify factors influencing conversion from laparoscopic to open surgery. Variables included in the model were selected based on clinical relevance and statistical significance in univariate analyses. These variables included the severity of the condition according to the Tokyo criteria, the Charlson Comorbidity Index (CCI), and other relevant demographic and clinical factors. *p* < 0.05 was considered statistically significant.

## Results

Based on data comprising 6,468 patients, the overall incidence of gallbladder perforation at our clinic was 1.26%. After screening patients for eligibility against the study criteria, we identified 42 who met our criteria. Of all patients undergoing gallbladder surgery, 0.64% had type I gallbladder perforations according to the modified Niemeier classification. The 42 patients included in this study had a median age of 68 (55–85) years, with 38.1% being female. Additionally, 42.90% had a known history of calculi before surgery. At their first presentation to the Emergency Department, 34 patients complained of abdominal pain, 18 of vomiting, and 16 had a fever (> 38 °C). Regarding preoperative comorbid conditions, 42.9% of the patients had diabetes mellitus, 61.9% had hypertension, 52.4% had coronary artery disease, 19.0% had cerebrovascular disease, and 19.0% had chronic lung disease. The preoperative radiologic diagnosis was determined preoperatively using ultrasonography for 38.1%, CT for 52.4%, and MRI for 9.5%. The median gallbladder wall thickness was 14 millimeters. In 4 patients imaged with MRI, the median bile duct diameter was 11 millimeters, and of these, 2 had fundus perforation and 1 had body perforation. Next, we compared patients based on their surgical procedure. Surgery was started laparoscopically in all patients. Patients were grouped based on whether their surgery was completed laparoscopically (LC; *n* = 28) or converted to open surgery (COC; *n* = 14). Conversions were performed reactively due to intraoperative complications (2 bleeding, 1 bile duct injury) in 21.4% of cases, and preemptively (surgeon’s prediction of difficulty and failure to provide a critical view of safety) in 78.5% of cases. The age distribution did not differ significantly between the LC group (71.50 (55–85) years) and the COC group (68 (59–83) years; *p* = 0.218). However, the sex distribution did differ significantly between the LC group (female: male ratio = 14:14) and the COC group (2:12; *p* = 0.025). The preoperative serum WBC counts and glucose values did not differ significantly between the LC and COC groups. The Charlson comorbidity index was evaluated, with Charlson comorbidity index II (35.3%) being most common in the LC group, and Charlson comorbidity index IIIB (38.5%) in the COC group (*p* = 0.011). The Tokyo classification criteria showed class II (57.1%) was most common in the LC group, while class III (71.4%) was observed in the COC group (*p* = 0.007; Table 1).

**Table 1:** Preoperative clinical characteristics of patients, comparison of laparoscopic cholecystectomy and conversion to open cholecystectomy groups

	Total (*n* = 42)	LC group	COC group	*p* = value
		(*n* = 28, 66.6%)	(*n* = 14, 33.3%)	
Age, median (range), year	68 (55–85)	71.50 (55–85)	68 (59–83)	0.218*
Sex, F:M, n	16:26	14:14	2:12	0.025**
History of calculi before perforation, n (%)	18 (42.9%)	13 (46.4%)	5 (35.7%)	0.508**
Modes of diagnosis, n (%)				0.161**
US	16 (38.1%)	12 (42.9%)	4 (28.6%)	
CT	22 (52.4%)	15 (53.6%)	7 (50.0%)	
MRI	4 (9.5%)	1 (3.6%)	3 (21.4%)	
Wall thickness, median, milimeter	14 (7–16)	14 (8–16)	13 (7–14)	0.681*
Presenting complaint, n (%)				
Abdominal pain	34 (80.95%)	24 (85.7%)	10 (71.4%)	0.266**
Vomiting	18 (42,85%)	11 (39.3%)	7 (50.0%)	0.508**
Fever	16 (38.09%)	12 (42.9%)	4 (28.6%)	0.369**
Comorbidities, n (%)				
Diabetus Mellitus	18 (42.9%)	10 (35.71%)	8 (57.14%)	0.186**
Hypertension	26 (61.9%)	17 (60.71%)	9 (64.28%)	0.822**
Coronary artery disease	22 (52.4%)	16 (57.14%)	6 (42.85%)	0.382**
Cerebrovascular disease	8 (19.0%)	5 (17.85%)	3 (21.42%)	0.781**
Chronic lung disease	8 (19.0%)	5 (17.85%)	3 (21.42%)	0.781**
ASA, n (%)				0.392**
I	1 (2.4%)	1 (3.6%)	0	
II	6 (14.3%)	3 (10.7%)	3 (21.4%)	
III	24 57.1%)	18 (64.3%)	6 (42.9%)	
IV	11 (26.2%)	6 (21.4%)	5 (35.7%)	
CCI, median (IQR)	2 (1–6)	2 (1–3)	3 (1–6)	0.02*
Tokyo classification, n (%)				0.007**
I	7 (16.7%)	6 (21.4%)	1 (7.1%)	
II	19 (45.2%)	16 (57.1%)	3 (21.4%)	
III	16 (38.1%)	6 (21.4%)	10 (71.4%)	
Preoperative WBC, median (range)	12300 (8600–28920)	11900 (9800–24210)	12300 (8600–28920)	0.486*
Preoperative glucose, median (range)	146 (111–206)	153.50 (111–206)	146.0 (135–176)	0.789*

n: number, SD: standard deviation, F: female, M: male, US: Ultrasonography, CT: Computed tomografi, MRI: magnetic resonance imaging, WBC: white blood cell, MODS: Multiple Organ Dysfunction Sydrome, LC: laparoscopic cholecystectomy, COC: conversion to open cholecystectomy, CCI: Charlson comorbidity index, IQR: Interquartile range, ASA: American Society of Anaesthetiologist,^†^: Clavien – Dindo ≥ III

Bold values indicate statistically significant *p* values (*p* < 0.05)

*: Mann -Whitney U test, **: Chi-square test, ***: Fisher’s exact tests

The anatomically determined perforation area differed significantly between the LC and COC groups (*p* < 0.001). In the LC group, 22 patients had perforations in the fundus, 4 in the body, and 2 in the neck. In the COC group, 2 patients had perforations in the fundus, 4 in the body, and 8 in the neck (Table 2). There were significant differences in perforation rates between the fundus and body (*p* = 0.009) and between the fundus and neck (*p* < 0.001) but not between the body and neck (*p* = 0.180; Table 3).

**Table 2:** Comparison of intraoperative and postoperative clinical characteristics of laparoscopic cholecystectomy and conversion to open cholecystectomy groups

	Total (*n* = 42)	LC group	COC group	*p* = value
		(*n* = 28, 66.6%)	(*n* = 14, 33.3%)	
Perforation area, n (%)				< 0.001**
Fundus	24 (57.1%)	22 (78.5%)	2 (14.2%)	
Body	8 (19.0%)	4 (14.2%)	4 (28.5%)	
Neck	10 (23.8%)	2 (7.14%)	8 (57.1%)	
Completion of surgery, n (%)				0.040**
Total cholecystectomy	36 (85.7%)	24 (85.7%)	12 (85.7%)	
Subtotal cholecystectomy	2 (4.76%)	0	2 (14.3%)	
Cholecystostomy	4 (9.52%)	4 (14.3%)	0	
Modified Nassar scales, n (%)				0.040***
I	1 (2.6%)	1 (3.8%)	0	
II	11 (28.2%)	9 (34.6%)	2 (28.2%)	
III	15 (38.5%)	12 (46.2%)	3 (23.1%)	
IV	10 (25.6%)	4 (15.4%)	6 (46.2%)	
V	2 (5.1%)	0	2 (15.4%)	
No record	3	2	1	
Surgical complication, n (%)				0.346**
Negative	26 (61.9%)	20 (71.4%)	6 (42.9%)	
Positive	16 (38.0%)	8 (28.6%)	8 (57.1%)	
Wound site infection		0	2 (14.3%)	
Intrabdominal sepsis		2 (7.1%)	4 (28.6%)	
Pulmonary infection		4 (14.3%)	0	
MODS		2 (7.1%)	0	
Bile tract injury		0	2 (14.3%)	
Clavien – Dindo classification, n (%)	30	17	13	0.011**
I	7 (23.3%)	5 (29.4%)	2 (15.4%)	
II	6 (20.0%)	6 (35.3%)	0	
IIIA	3 (10.0%)	1 (5.9%)	2 (15.4%)	
IIIB	6 (20.0%)	1 (5.9%)	5 (38.5%)	
IV	3 (10.0%)	0	3 (23.1%)	
V	5 (16.7%)	4 (23.5%)	1 (7.7%)	
Major complication^†^, n	17/30	17-Jun	13-Nov	0.007**
Bile culture reproduction, n (%)				
Negative	37 (88.09%)	24 (85.72%)	13 (92.85%)	0.558**
Positive	5 (11.90%)	4 (14.28%)	1 (7.14%)	
Candida tropicalis				
Escherichia coli				
Duration of hospitalization, median (range), day	5 (2–25)	4.5 (2–19)	5.0 (3–25)	0.483*
Mortality, n (%)				0.65**
Positive	5 (11.9%)	4 (14.3%)	1 (7,14%)	
Negative	37 (88.0%)	24 (85.7%)	13 (92.9%)	
Duration of surgery, median (range), minutes	120 (60–225)	105.0 (60–225)	125.0 (110–180)	0.035*
Primary surgeon age, median (range), year	44 (34–63)	42 (34–63)	55 (36–59)	0.001*

n: number, WBC: white blood cell, MODS: Multiple Organ Dysfunction Sydrome, LC: laparoscopic cholecystectomy, COC: conversion to open cholecystectomy, Clavien – Dindo ≥ III

Bold values indicate statistically significant *p* values (*p* < 0.05)

*: Mann -Whitney U test, **: Chi-square test, ***: Fisher’s exact tests

**Table 3:** Distribution of gallbladder perforation areas according to groups

Perforation area, *n* (%)	LC group	COC group	*p* = value
Fundus Body	22 (84.6%) 4 (15.4%)	2 (33.3%) 4 (66.6%)	0.009*
Fundus Neck	22 (91.7%) 2 (8.3%)	2 (20.0%) 8 (80.0%)	< 0.001*
Body Neck	4 (66.6%) 2 (33.3%)	4 (33.3%) 8 (66.6%)	0.18*

n: number, LC: laparoscopic cholecystectomy, COC: conversion to open cholecystectomy

Bold values indicate statistically significant *p* values (*p* < 0.05)

*: Chi-square test

**Table 4:** Multivariate analysis of factors that may influence conversion from laparoscopic to open surgery

	B	*p*	OR	(95% CI)
Primary surgeon age	0.049	0.062*	1.05	0.98–1.12
Perforation area	0.916	< 0.001*	2.5	1.8–3.5
Sex	0.182	0.367*	1.2	0.8–1.8
CCI	0.336	0.038*	1.4	1.02–1.92
Tokyo classification	1.131	< 0.001*	3.1	2.1–4.5

p: value, CI: confidence interval, OR: Odds Ratio, CCI: Charlson comorbidity index

Bold values indicate statistically significant *p* values (*p* < 0.05)

*: Multivariate analysis

Total cholecystectomy was performed in 85.7% of patients in both the LC and COC groups, while subtotal cholecystectomy was performed in 14.3% of patients in the COC group and not at all in the LC group. The surgical procedure decisions did not differ significantly between groups (*p* = 0.040). Intraoperative findings, evaluated according to the Modified Nassar scales, showed class III (46.2%) was most common in the LC group, and class IV (46.2%) in the COC group (*p* = 0.04). Regarding surgical complications, 61.9% of the study cohort experienced no complications, while 38.1% developed complications. In the LC group, two patients developed intraabdominal sepsis, four developed lung infections, and two developed multiple organ dysfunction syndrome. In the COC group, two patients developed wound site infections, four developed intraabdominal sepsis, and two suffered biliary tract injuries. The incidence of surgical complications did not differ significantly between groups (*p* = 0.346). Postoperative complications, evaluated with the Clavien–Dindo classification, showed class II (35.3%) was most common in the LC group, and class IIIB (38.5%) in the COC group (*p* = 0.011). Major complications (Clavien – Dindo ≥ III) differed significantly between the LC and COC groups (*p* = 0.007). Five patients (11.9%) experienced *Candida tropicalis* and *Escherichia coli* growth in bile fluid cultures. The postoperative hospitalization duration did not differ significantly between the LC group (median = 4.5 days) and the COC group (median = 5.0 days; *p* = 0.483). Mortality did not differ significantly between groups (*p* = 0.650). The operation times differed significantly between the LC (median = 105.0 min) and COC groups (median = 125.0 min; *p* = 0.035). Additionally, the primary surgeon’s age differed significantly between the LC (median = 42 years) and COC groups (median = 55 years; *p* = 0.001; Table 2).

Multivariate analysis was performed evaluating male gender, surgeon age, perforation area, CCI, and Tokyo classification. Sex: OR = 1.2 (CI: 0.8–1.8), *p* = 0.367. This value indicates that women compared the surgical outcome with men and sex did not significantly influence the surgical outcome (*p* > 0.05). The OR value exceeds 1 and is referenced to men. Tokyo classification: OR = 3.1 (CI: 2.1–4.5), *p* < 0.001. This value indicates that the Tokyo II and Tokyo III classifications compared the surgical outcome with the Tokyo I classification, and the Tokyo classification significantly influenced the surgical outcome (*p* < 0.001). Tokyo I is taken as reference. Perforation area: OR = 2.5 (CI: 1.8–3.5), *p* < 0.001. This value compares the surgical outcome of body and neck perforations with fundus perforation, indicating that the perforation site significantly influenced the surgical outcome (*p* < 0.001). Fundus is taken as reference (Table 4).

## Discussion

Emergency surgical procedures for gallbladder perforation represent 2–10% of all gallbladder surgeries [19]. After analyzing data comprising 6,468 patients who underwent gallbladder surgeries at our clinic, we found a gallbladder perforation incidence of 1.26% (*n* = 82). Before classification, our incidence of perforation surgery was lower than in the literature. In the modified Niemeier gallbladder perforation classification, type I represents generalized biliary peritonitis. Patients with type I gallbladder perforations almost always require urgent surgical intervention [6, 7]. Among all patients undergoing gallbladder surgery, 0.64% (*n* = 42) had modified Niemeier type I gallbladder perforations, representing 51.21% of all gallbladder perforations. The incidence of type I gallbladder perforations was reported to be 8.6% by Gupta et al. [13], 49.6% by Sahbaz et al. [20], 60% by Rajput et al. [21], and 52.2% by Kumar et al. [22]. Since gallbladder perforations are rare, variable rates have been reported in the literature. Our study presented the results of our laparoscopic experience with patients with modified Niemeier type I gallbladder perforations requiring an emergency surgical procedure.

At their initial presentation to the Emergency Department, 34 (80.95%) patients complained of abdominal pain, 18 (42.85%) of vomiting, and 16 (38.09%) had a fever (> 38 °C). The presenting complaints of gallbladder perforations include abdominal pain, nausea, and vomiting [3]. Krishnamurthy et al. [11] found abdominal pain was the most common presenting symptom in 93.9% of patients. We also identified abdominal pain as the most common presenting symptom.

When we evaluated the comorbid conditions of the patients included in our study, we found that hypertension was the most common (61.9%), coronary artery disease was the second most common (52.4%), and diabetes mellitus was the third most common (42.9%). When the study groups were examined within themselves, no statistically significant difference was observed between the groups in terms of comorbid diseases. Krishnamurthy et al. [11] found diabetes mellitus in the first place (80.0%), hypertension in the 2nd place (60.0%), and ischemic heart disease in the 3rd place (33.3%) in the comorbid status evaluation of their population. Stefanidis et al. [23] found that the most common comorbid conditions in their study were cardiovascular diseases (50.0%) and diabetes mellitus (25.0%) in the 2nd place. Due to the rarity of gallbladder perforations, studies have a low patient population. Therefore, there are differences in the ranking of the most common comorbid conditions in the literature. However, as supported by our study, cardiac diseases, and diabetes mellitus are the most common comorbid conditions in gallbladder perforation. We decided to elaborate on the examination of comorbid data in the preoperative period and evaluated the ASA Physical status classification system and the Charlson comorbidity index. According to the ASA classification, the population was distributed as ASA I (2.4%), ASA II (14.3%), ASA III (57.1%), and ASA IV (26.2%). ASA III represented the most common group. Krishnamurthy et al. [11] also found the ASA III group to be the most common with 53.3%. Sahbaz et al. [20] found the ASAI group as the most common group in their study in which all perforation types were evaluated. Krishnamurthy et al. [11] evaluated surgically treated gallbladder perforations and found similar results to ours. LC (median 2 (1–3)) and COC (median 3 (1–6)) groups were evaluated by the Charlson comorbidity index (*p* = 0.02). When the literature was reviewed, the Charlson comorbidity index was not evaluated in gallbladder perforations. However, Ramírez-Giraldo et al. [24] found the index to be a median of 1.0 in easy cholecystectomies and a median of 2.0 in difficult cholecystectomies (*p* < 0.001). Although all gallbladder perforations can be classified as difficult surgery, it can be estimated that the COC group refers to the more difficult group. Our study also supported the literature in this respect. However, to provide accurate information, there is a need for contribution in this aspect of the literature.

Findings at the presentation were classified according to Tokyo Guidelines 2018. Class II (57.1%) was more common in the LC group, while Class III (71.4%) was more common in the COC group (*p* = 0.007). Ramírez-Giraldo et al. [24] found a significant association between difficult cholecystectomies and Tokyo Classification 2018. The components of the Tokyo Guidelines 2018, which classify the relationship with the severity of cholecystitis findings, were not evaluated in other studies relevant to our study. However, Tokyo Classification 2018 is associated with the components of studies evaluating the factors affecting difficult laparoscopic cholecystectomy in the literature. These studies found a statistically significant association of components with difficult cholecystectomy [17, 25–29]. In our study, we showed that more severe cholecystitis findings were observed in the COC group with the Tokyo Classification 2018.

Laparoscopy was the first choice for emergency surgery in all patients included in our study. We evaluated the feasibility of minimally invasive surgery by dividing the patients into two groups (LC and COC). Krishnamurthy et al. [11] (86.8%) and Xaio et al. [12] (60%) found the most common perforation site to be the fundus. The fundus was also the most common perforation site in our study (57.1%). This observation has been attributed to low blood flow in the fundus in the literature. When the feasibility of minimally invasive surgery was evaluated according to the perforation location, there were significant differences between the fundus (*n* = 24) and the trunk (*n* = 8; *p* = 0.009) and between the fundus and the neck (*n* = 10; *p* < 0.001) but not between the body and the neck (*p* = 0.18). We concluded that the closer the gallbladder perforation site was to Calot’s triangle, the higher the likelihood of conversion to open surgery. The most common cause of conversion in gallbladder surgery is the inability to perform safe surgery due to adhesions in Calot’s triangle [30]. Due to the inflammatory process in gallbladder perforation, we believe that adhesions increase with proximity to Calot’s triangle, with a concomitant increase in the possibility of termination of minimally invasive surgery.

We found no significant difference in complications between groups (*p* = 0.346). When we searched the literature, we found no studies with sufficient patients to evaluate only type I gallbladder perforations. Therefore, we compared all gallbladder perforations with type I. The most common morbidity in our study was intraabdominal sepsis (14.2%). Zhang et al. [1] evaluated the incidence of postoperative complications, finding that 4.5% of patients developed wound infections, 2.3% developed paralytic ileus, 2.3% developed heart failure and acute respiratory distress syndrome, and 2.3% developed renal failure. Sahbaz et al. [20] identified wound infection as the most common morbidity in all types (5.26%). The literature shows that the most common morbidity in all types of gallbladder perforation is wound infection. We attribute the fact that the most common morbidity in our study was not wound infection to the feasibility of minimally invasive surgery. Complications were classified according to the Clavien-Dindo classification. Clavien-Dindo II (*n* = 6 (35.3%)) was more common in the LC group and Clavien-Dindo IIIB (*n* = 5 (38.5%)) in the COC group (*p* = 0.011). Ramírez-Giraldo et al. [24] compared easy and difficult laparoscopic cholecystectomies and found a relationship between the groups according to the Clavien-Dindo classification. In our study, all cholecystectomies were performed due to gallbladder perforation. So actually all surgical procedures can be considered as difficult. Therefore, it was expected that there would be no statistical relationship between the groups. Major complications (Clavien-Dindo ≥ III) were evaluated in the LC (*n* = 6) and COC (*n* = 11) groups (*p* = 0.007). The higher incidence of major complications in the COC group may be explained by the minimally invasive nature of the surgery and intraoperative difficulty. Modified Nassar scales were used to evaluate intraoperative surgical difficulty. Modified Nassar scales III in the LC group (*n* = 12 (46.2%)) and IV (*n* = 6 (46.2%)) were more frequent in the COC group. It was not surprising that major complications were more common in the COC group, which was more difficult in terms of intraoperative findings.

We evaluated the postoperative hospitalization duration in the LC (median = 4.5 days, range = 2–19) and COC (median = 5.0 days, range = 3–25) groups. Rajput et al. [21] performed open surgery for gallbladder perforations and reported a mean hospitalization duration of 12 days for patients with type I gallbladder perforations. We found that it was shorter with the laparoscopic approach (*p* = 0.483). The mortality rate in our study cohort was 11.9% (*p* = 0.650). Sahbaz et al. [20] reported a mortality rate of 8.27% for all gallbladder perforations. While we found a significant difference in surgical procedure duration between groups, we believe this difference reflects the preparation process during the transition from laparoscopic to open surgery.

The age of the primary surgeon correlated significantly with the completion of minimally invasive surgery in our study (*p* = 0.001). Younger surgeons were able to rectify gallbladder perforations with laparoscopic surgery. A review of the literature showed varying regarding minimally invasive surgery. While some studies indicated that the learning curve is superior in the younger age group, others have demonstrated the success of the older group in minimally invasive surgery due to experience [30–32]. Another important detail at this point is to evaluate whether young surgeons lack experience in the transition to open surgery. In our study, there was no statistical difference between the groups in terms of complications. Larger series are needed to evaluate this point. We also think that the age of the surgeon should be included in the evaluation criteria in studies evaluating difficult cholecystectomies [33, 34].

Five (11.9%) patients had *Candida tropicalis* and *Escherichia coli* growth in the intraoperative bile fluid cultures. We did not find any comparable data in the literature on gallbladder perforation. In a study evaluating patients who underwent laparoscopic surgery for acute cholecystitis, there was a much higher growth rate [35]. We think that the low growth rates in bile cultures in our study may be due to the small number of patients and the broad-spectrum empiric antibiotic treatment applied due to the preoperative diagnosis of gallbladder perforation.

Our study demonstrates the results of minimally invasive surgery in treating modified Niemeier type I gallbladder perforations. Its limitations include its retrospective design and small sample size due to the condition’s rarity. Additionally, only patients with gallbladder perforation detected on preoperative imaging and who underwent minimally invasive surgery with this diagnosis were included in this study. The exclusion of patients with gallbladder perforation detected intraoperatively and patients who underwent direct open surgery may affect the results of the study. However, our results show that the minimally invasive approach in gallbladder perforation reduces complications, and hospital stay.

## Conclusion

Minimally invasive surgery is a feasible and effective method for treating modified Niemeier type I gallbladder perforations. We believe that the surgical procedure should be started laparoscopically in patients with a prediagnosis of gallbladder perforation.

## Data Availability

The database of this study is open to sharing. It can be obtained from the authors upon request.

## Declarations

### Scientific responsibility statement

The authors declare that they are responsible for the scientific content of the article, including study design, data collection, analysis and interpretation, writing, part or all of the outline, preparation and scientific review of the content, and approval of the final version of the article.
